# Carotid interventions and evidence-based medicine: not always an easy marriage

**DOI:** 10.1093/bjs/znab038

**Published:** 2021-02-14

**Authors:** A Ross Naylor

**Affiliations:** Leicester Vascular Institute, Glenfield Hospital, Leicester UK

‘Never find your delight in another’s misfortune’

Publilius Syrus (85–43BC)

If ever a word encapsulated four decades of practice regarding the management of carotid disease, it would have to be ‘schadenfreude’. In 1951, Miller Fisher reported that emboli from extracranial carotid stenoses caused ischaemic stroke and, within 3 years, a surgical strategy had been developed for removing this embolic source from the carotid bifurcation. Over the next two decades, indications for carotid endarterectomy (CEA) were driven by intuitive reasoning, and CEA became one of the most commonly performed vascular procedures across the world.

Surgeons of that era were not, however, used to non-vascular professionals scrutinizing their practice, and they did not take kindly to neurologists suggesting that some CEA indications might be inappropriate and that complication rates in the real world were not as good as the doyens of vascular surgery would have them believe. In the 1980s, the conflict between surgeon enthusiasts and sceptical neurologists became the catalyst for the dawn of evidence-based practice in vascular surgery (now taken for granted), with RCTs being used to answer important clinical questions. Two landmark RCTs saw CEA secure a proven role in the management of symptomatic patients with 50–99 per cent stenoses. Schadenfreude overflowed, but this was not limited to the surgeon’s joy at ousting those troublesome neurologists. It was also voiced by non-participating surgeons who played the ‘I told you so’ card and who still remained disinclined to accept that RCTs should inform future decision-making, rather than intuitive reasoning.

Having secured level 1 evidence supporting CEA, any hopes that vascular surgeons harboured that they could return, unchallenged, to their old ways were permanently dashed with the emergence of carotid artery stenting (CAS) during the early 1990s. In every other aspect of surgical practice, less invasive techniques were replacing open surgical procedures, but not where the carotid artery was concerned. If surgeons thought their skirmishes with the neurologists were difficult, these paled into insignificance with the growing prominence of interventional radiologists and cardiologists who actively promoted CAS over CEA. CAS practitioners were driven by a much more powerful medical industrial complex and they did not feel constrained by rules established by the recently victorious surgeons. Unfortunately, CAS practitioners then repeated exactly the same mistakes that the surgeons had committed two decades earlier, and debates about CEA *versus* CAS were characterized by conflict, mistrust, schadenfreude, implausibly good results (on both sides), and a tendency to shoot the messenger should new RCT data fall foul of one of the warring disciplines.

No fewer than 22 RCTs, involving almost 10 000 patients, have now compared CEA with carotid angioplasty or CAS^1^. Unfortunately, many were too small, some stopped early on a random low or high, and at least one was prohibited from publishing poor CAS results on the orders of the sponsoring stent company. Moreover, a trend emerged whereby, should a randomized trial publish outcomes that pronounced victory for one side over the other, the losing discipline invariably claimed that there were serious methodological flaws (never raised when the methodology paper was published) or that newer technologies now rendered the data obsolete, so that the results could, therefore, be ignored.

Notwithstanding these difficulties, several important observations were made. First and foremost, meta-analyses clearly showed that, once the 30-day perioperative period had elapsed, 5- and 9-year rates of ipsilateral stroke were virtually identical, that is CAS was as durable as CEA^1^. However, meta-analyses also showed that the 30-day death/stroke rate was significantly higher after CAS than CEA^1^. This is why contemporary guidelines still confer a higher level of evidence supporting CEA, compared with CAS^2^. Accordingly, it is the predicted magnitude of the 30-day risk that will largely determine the choice of CEA or CAS in individual patients in the future.

So, what challenges remain? First is the need to validate clinical and/or imaging features that predict a higher or lower 30-day risk of death/stroke after CEA or CAS, as this will greatly inform individual patient consent. To date, secondary analyses from the 22 RCTs suggest that CEA is probably safer than CAS in symptomatic patients aged over 70 years, where interventions are undertaken within 7–14 days of symptom onset, and in situations where the predicted stroke risk after CAS is higher (such as segmental/remote plaques, plaque length over 13 mm, and a heavy burden of pre-existing white matter lesions in the brain)^1^. Of these, the single most important is the risk associated with intervening within 7–14 days of symptom onset. This is the interval when the patient is most likely to suffer recurrent stroke, and guidelines^2^ now recommend that carotid interventions should be undertaken during this high-risk period. At present, meta-analyses of RCT data suggest that CEA is safer than CAS when performed within 7–14 days of symptom onset^1^. It has been suggested that a new CAS innovation, transcarotid artery revascularization (TCAR), may provide outcomes similar to CEA, but TCAR proponents have never published outcome data for procedures performed within 7–14 days of symptom onset. This is essential information for the future as, without it, guidelines are unlikely to change their recommendations.

Other challenges include the intense debate about whether reductions in annual stroke rates in asymptomatic patients on modern medical therapy mean that fewer patients might now benefit from CEA/CAS (generating considerable neurologist schadenfreude once again). This is being tested in CREST-2, which is likely to be the last major RCT to compare CEA/CAS with best medical therapy in asymptomatic patients. It is unlikely that the RCTs including symptomatic patients will be repeated, mainly because of the greatly increased benefit conferred by intervening in the first 7–14 days after symptom onset. There is currently no equipoise for randomizing patients in this ‘high-risk for stroke’ period, which is when most carotid interventions are now performed. Although carotid stenosis of at least 50 per cent is present in one in five patients presenting with stroke or transient ischaemic attack^3^, the UK National Vascular Registry^4^ has reported a 25 per cent decline in annual CEA numbers in symptomatic patients over the past decade. This is most likely due to a proportional reduction in ischaemic strokes owing to thromboembolism from extracranial carotid stenoses, possibly because a greater proportion of patients with cardiovascular disease are now prescribed better antiplatelet and statin therapy than before.

There are also important clinical governance issues to be addressed, not least the fact that CAS practitioners in the USA perform fewer than two CAS interventions on average per year^5^, which may account for some specialties reporting 30-day death/stroke rates of 6 per cent in asymptomatic patients (twice the accepted threshold), without anyone acknowledging that, at this level of risk, interventions are harmful rather than beneficial to the patient. Another controversial issue is the relationship between asymptomatic carotid stenosis and cognitive dysfunction. Evidence suggests that there is an association, but this may simply mean that both conditions share similar risk factors. Given the controversy about the optimal management of asymptomatic patients (in general), it would be inappropriate to expand indications uncritically to include preventing cognitive decline without better evidence.

In the past, surgeons and interventionists have been guilty of promoting a ‘one victor only’ concept, whereas it is clear that CEA and CAS will evolve complementary roles. It is essential, however, that these roles are determined by evidence and collaboration, rather than by returning to a bygone era of intuitive reasoning, turf wars, and schadenfreude.

*Disclosure*. The author declares no conflict of interest.
